# Ebola virus disease in pregnancy: a systematic review and meta-analysis

**DOI:** 10.1093/trstmh/trab180

**Published:** 2021-12-02

**Authors:** Nzelle D Kayem, Charlotte Benson, Christina Y L Aye, Sarah Barker, Mariana Tome, Stephen Kennedy, Proochista Ariana, Peter Horby

**Affiliations:** Nuffield Department of Medicine, University of Oxford, Oxford OX3 7LG, UK; Women's Centre, John Radcliffe Hospital, Oxford University Hospitals, Oxford OX3 9DU, UK; Women's Centre, John Radcliffe Hospital, Oxford University Hospitals, Oxford OX3 9DU, UK; Nuffield Department of Women's and Reproductive Health, University of Oxford, Oxford OX3 9DU, UK; Women's Centre, John Radcliffe Hospital, Oxford University Hospitals, Oxford OX3 9DU, UK; Nuffield Department of Women's and Reproductive Health, University of Oxford, Oxford OX3 9DU, UK; Nuffield Department of Women's and Reproductive Health, University of Oxford, Oxford OX3 9DU, UK; Nuffield Department of Medicine, University of Oxford, Oxford OX3 7LG, UK; Nuffield Department of Medicine, University of Oxford, Oxford OX3 7LG, UK

**Keywords:** case fatality rate, Ebola, maternal outcomes, perinatal outcomes

## Abstract

This review synthesises and appraises evidence on the effects of Ebola virus disease (EVD) in pregnancy. We searched bibliographic databases from dates of inception to November 2020, yielding 28 included studies.

The absolute risk of maternal death associated with EVD was estimated at 67.8% (95% confidence interval [CI] 49.8 to 83.7, I^2^=85%, p<0.01) and the relative risk of death in pregnant women compared with non-pregnant women was estimated at 1.18 (95% CI 0.59 to 2.35, I^2^=31.0%, p=0.230). The absolute risk for foetal losses was estimated at 76.9% (95% CI 45.0 to 98.3, I^2^=96%, p<0.01) and neonatal death was 98.5% (95% CI 84.9 to 100, I^2^=0.0%, p=0.40). The gap analysis suggests limited or no data on the clinical course, non-fatal perinatal outcomes and EVD management in pregnant women. The review suggests that EVD has a high maternal and perinatal mortality, underscoring the urgent need for preventative and therapeutic solutions and improved screening and follow-up of pregnant women and newborns during outbreaks. There is not enough evidence to conclusively rule out pregnancy as a risk factor for mortality and there is limited evidence on the disease course, outcomes and management of EVD in pregnancy, and this supports the need for robust clinical trials and prospective studies that include pregnant women.

## Introduction

Ebola virus disease (EVD) is caused by Ebola virus and was first reported in South Sudan and along the Ebola River in the Democratic Republic of Congo (DRC) in 1976.^1^ EVD occurs sporadically, with all known outbreaks originating in sub-Saharan African (SSA), where an estimated 22 million people in 22 West and Central African countries are at risk of infection.^2^ The reservoir host for Ebola virus is thought to be the fruit bat.^1^

EVD has an abrupt onset with an incubation period of 2–21 d.^1^ Clinical features range from non-specific symptoms to multisystem involvement with haemorrhagic manifestations and multiorgan failure. Disease outbreaks in humans have so far been due to three viral species: *Zaire ebolavirus* (EBOV), *Bundibugyo ebolavirus* (BDBV) and *Sudan ebolavirus* (SUDV).^3^

A meta-analysis evaluating EVD mortality in the general population estimated a pooled crude case fatality rate of 65.0% (95% confidence interval [CI] 54.0 to 76.0, I^2^=97.98%).^4^ Suggested reasons for the high amounts of study heterogeneity were study variability, differences in outbreaks and variability in outbreak responses.^4^ In the DRC, the estimated crude case fatality rate for confirmed and probable cases was 66% in the 2018–2020 Kivu/Ituri Ebola outbreak and 42.3% in the 2020 Équateur outbreak.^5,6^ The improvements seen in the Équateur outbreak were likely due to the availability of vaccines and drugs.^7^

The prognosis of EVD is thought to be very poor in pregnant women, with maternal case fatality rates ranging from 74 to 100% and an aggregated maternal mortality of 84.3% in one review,^8^ 86% in another^9,10^ and 72% in a more recent review.^11^ Based on these reviews, foetal losses ranged from 23 to 100%^9,10^ and neonatal losses were frequent at 99.1–100%.^9,10^ While there are several reviews summarising evidence on EVD in pregnancy, the evidence is often aggregated or fails to account for differences in sample sizes and study variability. This is important because a quantitative synthesis that takes into account variability and samples size differences is likely to provide a more reliable summary estimate and specify areas of potential bias to facilitate further enquiry or formulation of hypotheses for future research.^12^

The goal of the review was to critically appraise and summarise evidence on the effects of EVD in pregnancy and to identify current gaps in the evidence reported in the peer-reviewed and grey literature. The review specifically appraises and synthesises evidence on the clinical characteristics, the maternal and perinatal outcomes of EVD during pregnancy and the clinical management of maternal Ebola virus infection.

## Methods

The review was conducted based on the Preferred Reporting Items for Systematic Review and Meta-analysis (PRISMA) guidelines for conducting systematic reviews.^13^ The review was conducted as part of a broader review on the effects of the viral haemorrhagic fevers in pregnancy and the methods have previously been published.^14^

### Search strategy and selection criteria

In summary, various bibliographic databases were searched in December 2017 and references from relevant reviews and included studies were also searched. There were no language restrictions (translation software was used where appropriate to facilitate screening and data extraction). Updated searches were conducted in June 2018, September 2019 and November 2020. Databases searched included PubMed, Web of Science, Excerpta Medica database (Embase), World Health Organization (WHO) Global Health Library (WHOGHL), Cumulative Index to Nursing and Allied Health Literature (CINAHL), Cochrane Central Register of Controlled Trials, Cochrane Pregnancy and Childbirth Register, Cochrane Infectious Diseases Register, International Standard Randomised Controlled Trial Number (ISRCTN) Registry, WHO International Clinical Trials Registry platform, European Union (EU) Clinical Trials Register, Pan African Clinical Trials Registry and ClinicalTrials.gov. A combination of MeSH terms and keywords were used to capture pregnancy outcomes, clinical features and management of EVD in pregnancy from both published and grey literature (see Supplementary Table 1). The review was registered in the International Prospective Register of Systematic Reviews (PROSPERO) of the University of York and the National Institute for Health Research, under protocol CRD42018097022.

### Data extraction

Citations were screened using Rayyan, an online systematic review program.^15^ Screening of titles and abstracts, assessment of full text for eligibility and risk of bias assessment were performed independently by two of the reviewers (NDK, CB, CA, MT or SB). Each article was preassigned randomly to any two of these reviewers and any discrepancies were resolved through discussion or with another (third) reviewer.^14^

Studies were included if they presented data on EVD in pregnancy. All study designs, as well as original reports, briefs, letters, editorials and comments, were considered for inclusion. Exposure was defined by clinical or laboratory criteria. Outcomes were broadly classified as clinical characteristics, maternal and perinatal outcomes and management practices. Outcomes were not predefined and definitions were extracted where available, from each citation. Data were extracted from eligible studies and odds ratios (ORs) or proportions were estimated depending on the available information.^14^ Meta-analyses were performed if two or more studies reported on the same outcome and included at least five pregnant women or live births.^14^ This was a pragmatic decision given that the disease is rare and we are evaluating pregnant women, who are a population that is often excluded from clinical trials or research.^16^ Gestational ages were inconsistently reported. As such, all foetal deaths occurring before or during labour and delivery, including miscarriages, intrauterine foetal death and stillbirths, were labelled as foetal loss. Maternal death was defined as death of the pregnant woman during pregnancy, labour and delivery or within 1 month after delivery. Neonatal death was defined as death of a newborn within 1 month of birth.

### Statistical analyses

All statistical analyses were done using R version 4.0.2 (R Foundation for Statistical Computing, Vienna, Austria).^17^ The metaprop command was used for the proportional meta-analysis and the metabin command was used to summarise the effect estimate for ORs because they implement procedures specific to binary outcome data. The weighted summary proportion was calculated using the Freeman–Tukey double arcsine transformation.^18–20^ A random effects model was used and Cochran's Q, τ^2^ and Higgins I^2^ were calculated. The degree of heterogeneity was interpreted as none (I^2^<25%), low (I^2^=25–49%), moderate (I^2^=50–74%) or high (I^2^≥75%).^21^

### Sensitivity analysis and meta-regression

Post hoc sensitivity analyses were performed where possible, dropping studies with 0% or 100% proportions and studies with <10 pregnant women to assess if excluding studies with smaller sample sizes and with extreme proportions results in a statistically significant difference in summary estimates.^14^ Where I^2^ was >50%, meta-regressions were conducted to evaluate reasons for the observed heterogeneity. The explanatory variables evaluated were study design (cohort vs non-cohort), year in which outbreak occurred (before 2000 vs after 2000), sample size (<10 vs ≥10)^14^ and country.

### Publication bias

Peter's test was used in combination with funnel plots to assess for publication biases.^22^ Funnel plots were derived using a mixed effects model with sample size as the predictor and the double arcsine transformed proportion.^14^

### Risk of bias assessment

Risk of bias assessment was based on Murad et al.’s^23^ quality tool for case reports and case series studies, while the Newcastle–Ottawa scale was used for all cohort-type studies.^24^ Although aggregated scores are provided, the risk of bias was colour-coded to allow for a better interpretation of the study quality by readers, given that an aggregated score fails to highlight where specific weaknesses in the reported study design are found.^25^

### Gap analysis

Research gaps were identified using Robinson et al.’s framework,^26^ which allows not only for identification of gaps, but the reasons for the gaps. Gaps are classified under four categories: insufficient or imprecise information, biased information, inconsistency or unknown consistency and not the right information. Given that EVD is a rare disease and the selected population (pregnant women) is rarely included in clinical research,^16^ and given that a meta-analysis will not be possible for all outcomes, we modified the framework such that evidence on meta-analyses were analysed separately. As such, where Robinson et al.^26^ discuss gaps based on four categories, we had five categories: separating the options for insufficient and imprecise information into two distinct categories, for each objective we presumed certain outcomes and, as such, we assessed the evidence or lack thereof based on these objectives (detailed in a previous manuscript^14^).

## Results

**Figure 1.**
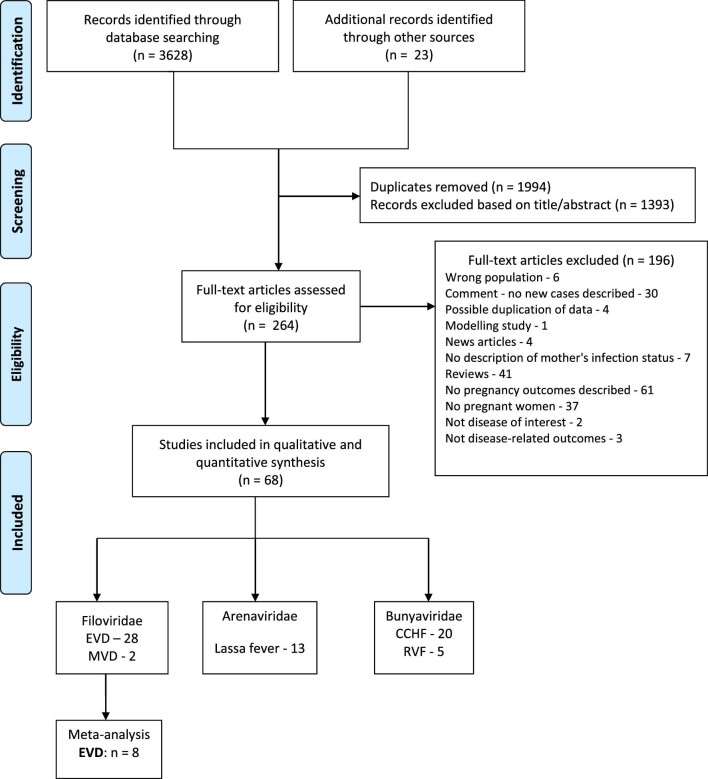
PRISMA flow diagram of selected studies. n: number of papers; CCHF: Crimean–Congo haemorrhagic fever; MVD: Marburg virus disease; RVF: Rift Valley fever.

A search of the databases yielded a total of 3651 records that were screened. A total of 264 full texts were evaluated for eligibility and 68 met the inclusion criteria (Figure 1). Twenty-eight of these were on EVD in pregnancy,^27–54^ and the characteristics of included studies are summarised in Table 1.

**Table 1.** Characteristics of studies reporting EVD in pregnancy

Author, year, country	Enrolment period	Study design	Cases—definition	Controls—definition	Number of pregnant women with EVD	Ebola virus strain, status of cases and diagnostic tests used	Gestational age estimation methods	Methods used to correct for confounding
Akerlund et al., 2015, Liberia	NS	Case report	An asymptomatic pregnant woman who tested positive for EBOV	NA	1	Zaire Confirmed PCR	NS	NA
Arias et al., 2016, Sierra Leone	June 2015	Case series	Cluster of EVD cases	NA	1	Zaire Confirmed PCR	NS	NA
Baggi et al., 2014, Guinea	June 2014	Prospective case series	Confirmed EVD in pregnant patients who presented to ETU	NA	2	Zaire Confirmed PCR	NS	NA
Baize et al., 2014, Guinea	28 February 2014–20 March 2014	Retrospective case series	Ebola-positive patients hospitalized in Gueckedou Macenta and Kissidougou	NA	1	Zaire Confirmed PCR	NS	NA
Baraka et al., 2019, DRC	NS	Case report	Neonate born to mother diagnosed with EVD	NA	1	Zaire Confirmed PCR	Self-report	NA
Bower et al., 2016, Sierra Leone	NS	Retrospective case report	PCR-negative but IgM/IgG-positive pregnant patient who delivered a PCR-positive stillborn infant	NA	1	Zaire Confirmed PCR, ELISA IgM and IgG	NS	NA
Bwaka et al., 1999, DRC	14 April–21 June 1995	Prospective case series	Patients with clinical case definition for EVD and/or ELISA Ag or IgM positive	NA	3	Zaire Clinical case definition ELISA Ag and IgM	NS	NA
Caluwaerts, 2015^a^, Guinea, Sierra Leone and Liberia	NS	Retrospective case series	Pregnant women with EVD who survived	NA	33	Zaire Confirmed PCR	NS	NA
Caluwaerts et al., 2016^a^, Guinea, Sierra Leone and Liberia	9 September 2014–17 April 2015	Retrospective case series	Pregnant cases of EVD with a negative blood test after clinical cure but positive amniotic fluid	NA	2	Zaire Confirmed PCR	NS	NA
Caluwaerts S. et al. 2018 Guinea, Liberia, Sierra Leone	23 March 2014–23 October 2015	Retrospective cohort	EVD-positive pregnant patients	EVD-positive non-pregnant women of reproductive age	77	Zaire Confirmed PCR	Fundal height	NS
Chertow et al., 2014, Liberia	23 August 2014–4 October 2014	Retrospective cohort	Patients with suspected EVD in the largest ETU in Monrovia, Liberia	NS	4	Zaire Confirmed and probable PCR	NS	NS
Dornemann et al., 2017, Guinea	NS	Case report	Pregnant woman diagnosed with EVD who delivered a live baby	NA	1	Zaire Confirmed PCR	Self-report, estimated at birth	NA
Dunn et al., 2016, Sierra Leone	October 2014	Case series	Cluster of cases from maternal index case	NA	1	Zaire Confirmed PCR	NS	NA
Francesconi et al., 2003, Uganda	12 September 2000–29 October 2000	Retrospective cross-sectional	Confirmed and probable EVD cases	Apparently healthy contacts of the case patients	1	Sudan Confirmed and probable ELISA Ag and IgG	NS	Multivariable logistic regression
Henwood et al., 2017, Liberia and Sierra Leone	15 September 2014–15 September 2015	Retrospective cohort	Pregnant women of reproductive age suspected of EVD	Non-pregnant women of reproductive age suspected of EVD	13	Zaire Confirmed PCR	Self-report, LNMP	Multivariable logistic regression
Khan et al., 1999, DRC	6 January 1995–16 July 1995	Mixed cohort	Clinically diagnosed or confirmed cases of EVD including the index case for the epidemic	NS	8	Zaire Confirmed, suspected and probable ELISA IgM and IgG	NS	NS
Lyman et al., 2018^b^, Sierra Leone	29 June 2014–20 December 2014	Mixed cohort	EVD-positive pregnant women presenting to ETU	EVD-negative and pregnant women presenting to ETU	67	Zaire Confirmed PCR	Self-report, case notes	Logistic regression
Mpofu et al., 2019^b^, Sierra Leone	29 June 2014–20 December 2014	Mixed cohort	EVD-positive pregnant women who presented to ETU with clinical case definition of EVD	EVD-negative pregnant women presenting with clinical case definition to ETU	55	Zaire Confirmed PCR, ELISA IgM and IgG	Self-report, case notes	Logistic regression
Muehlenbachs et al., 2016, Uganda and DRC	2000–2012	Retrospective case series	EVD-positive pregnant women	NA	2	Bundibugyo and Sudan Confirmed PCR, ELISA Ag and/or IgM	Self-report, estimated at birth	NA
Mupapa et al., 1999, DRC	April 1995–June 1995	Retrospective cohort	Pregnant women with EHF based on case definition	NS	15	Zaire Clinical case definition	LNMP, clinical examination	NS
Oduyebo et al., 2015, Sierra Leone	September 2014	Retrospective case report	EVD pregnant woman	NA	1	Zaire Confirmed PCR	Self-report	NA
Okoror et al., 2018, Sierra Leone	NS	Case report	Pregnant woman presenting to ETU with no foetal movements	NA	1	Zaire Confirmed PCR	Self-report	NA
Palvin et al., 2020, Sierra Leone	November 2014–May 2015	Retrospective case series	Asymptomatic pregnant women with EVD admitted to ETU	NA	6	Zaire Confirmed PCR, ELISA IgM and IgG	Self-report	NA
Schieffelin et al., 2014, Sierra Leone	25 May–18 June 2014	Cohort	Patients who had an illness that met the definition for suspected Lassa fever or EVD	NS	1	Zaire Confirmed PCR	NS	NS
van Griensven et al., 2016, Guinea	17 February 2015–3 August 2018	Mixed NRCT	EVD-positive women who received matched convalescent plasma	EVD-positive women admitted and treated with supportive care in the 5 months preceding the trial (historical controls)	8	Zaire Confirmed and probable PCR	NS	Logistic regression
Wamala et al., 2010, Uganda	1 August 2007–20 February 2008	Retrospective cohort	EVD confirmed or probable cases	EVD laboratory negative	1	Bundibugyo Confirmed, suspected and probable PCR, ELISA Ag, IgM and IgG	NS	Multivariable logistic regression
Williams et al., 2015, Liberia	June 2014	Case series	Cluster of EVD cases	NA	1	Zaire Confirmed PCR	NS	NA
WHO, 1978, DRC	1 September 1976–24 October 1976	Retrospective cohort	318 cases of EVD	NS	82	Zaire Confirmed IFA	NS	NS

Ag: antigen; EHF: Ebola haemorrhagic fever; ELISA: enzyme-linked immunosorbent assay; ETU: Ebola treatment unit; IFA: immunofluorescence assay; IgM: immunoglobulin M; IgG: immunoglobulin G; LNMP: Last Normal Menstrual Period; NA: not applicable; NS: not stated; NRCT: non-randomized controlled trial.

a These studies were excluded from the meta-analysis because of possible risk of duplication with another included study.

b These studies include the same group of pregnant women, however, one reports on clinical characteristics while the other reports on pregnancy outcomes.

A total of 354 pregnant women with EVD were included in the studies. The ages of 113 pregnant women were reported and ranged from 13 to 40 y.^29–31,37,39–44,46,47,50,51^ Gestational ages were inconsistently reported in 111 pregnant women and ranged from the first trimester to term (≥37 weeks).^31,36,39,41,43–47,53,54^ Most articles (15/23) reported on EVD pregnant women from West Africa, specifically Sierra Leone, Liberia and Guinea (Table 1).

### Clinical characteristics and course of maternal Ebola virus infection

The clinical features of EVD in pregnancy were non-specific and recorded in 101 pregnant women in 15 studies.^27,29,31,32,36,37,39,42–45,47,51^ Four studies satisfied the criteria for inclusion into a meta-analysis.^39,42,44,47^ Clinical features are summarised in Table 2.

**Table 2.** Clinical characteristics of pregnant women with Ebola virus disease

Clinical features	n	N	Percentage	Weighted summary percentage (95% CI)]
Fever	79	100	79.0	86.1 (62.5 to 99.6), I^2^=77%
Asthenia	76	101	75.2	87.4 (67.1 to 99.4), I^2^=72%
Abdominal pain	68	100	68.0	80.3 (49.8 to 99.1), I^2^=84%
Headache	59	99	59.6	77.3 (42.9 to 99.0), I^2^=87%
Nausea and vomiting	54	99	54.5	55.8 (21.1 to 87.8), I^2^=87%
Myalgia and arthralgia	53	100	53.0	65.8 (20.8 to 98.6), I^2^=92%
Diarrhoea	52	100	52.0	63.9 (18.1 to 98.4), I^2^=93%
Anorexia	39	100	39.0	64.0 (10.2 to 100), I^2^=95%
Vaginal bleeding	36	99	36.4	44.9 (2.4 to 92.5), I^2^=94%
Disordered level of consciousness	25	99	25.3	ND
Sore throat	24	99	24.2	42.4 (0 to 98.3), I^2^=97%
Bleeding otherwise unspecified	24	99	24.2	26.1 (11.9 to 43.0), I^2^=49%
Chest pain	20	100	20.0	14.8 (0.4 to 40.0), I^2^=80%
Hiccups	17	99	17.2	35.4 (0.0 to 100), I^2^=98%
Conjunctivitis	15	99	15.2	ND
Apathy	14	99	14.1	ND
Seizures	7	99	7.1	ND
Back pain	7	99	7.1	ND
Cough	5	100	5.0	ND
Rash	2	99	2.0	ND
Jaundice	1	84	1.2	ND

The table summarises the clinical features of pregnant women with EVD in order of decreasing frequency.

ND: not done because only one study satisfied the meta-analysis criteria; n: number of pregnant women who presented with the symptom; N: total number of pregnant women in the analysis.

The clinical course of infection during pregnancy was not described and most of the information was obtained from case reports or series. The length of hospital stay (evaluated as the time from admission to either discharge or death) was reported in 32 pregnant women in 12 studies and ranged from 0 to 32 d.^27,29,31,36,37,39,43,45–48,50,54^ The time from illness onset to admission was 1–7 d in 19 pregnant women from five studies^29,36,39,43,45^ and the time from illness onset to treatment was 3–7 d recorded in four pregnant women in three studies.^29,36,45^

### Maternal outcomes of Ebola virus infection

**Figure 2.**
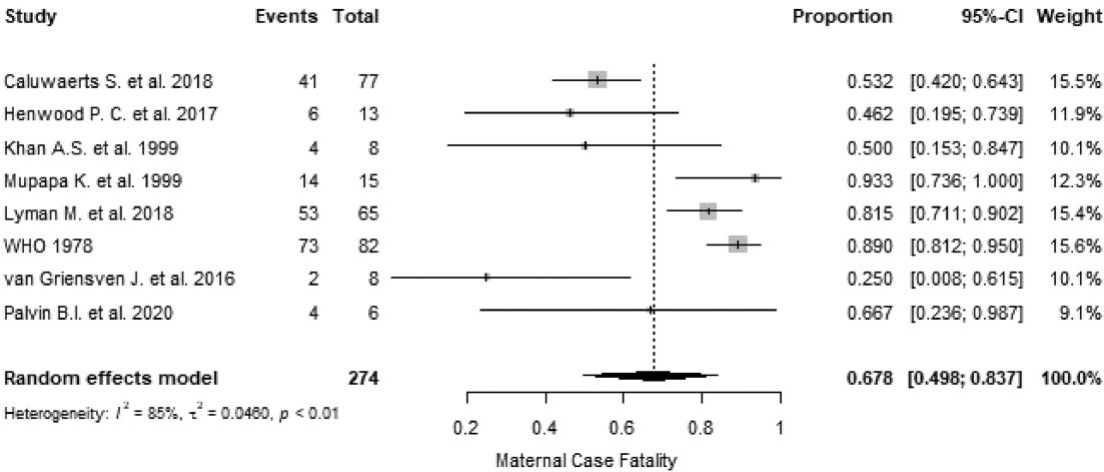
Proportional meta-analysis forest plot of studies reporting maternal death for EVD in pregnancy. p: p-value associated with Cochran's Q for heterogeneity; events: number of maternal deaths; total: total number of pregnant women included in the analysis.

The most-reported outcome in EVD-positive pregnant women was maternal death, with a weighted summary proportion of 67.8% (95% CI 49.8 to 83.7, I^2^=85%, p<0.01; Figure 2). The gestational age at which maternal death occurred was specified in 15 women and ranged from 14 weeks to term (≥37 weeks), with most deaths (9/15 [60.0%]) occurring in the third trimester.^36,39,44,47^

**Figure 3.**
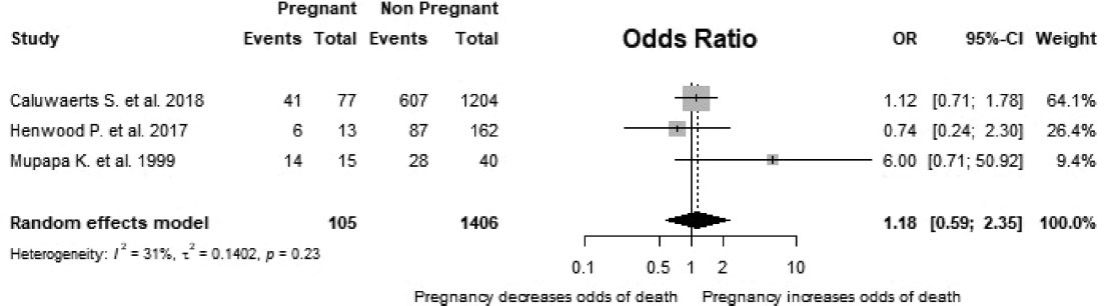
Forest plot showing the risk of death from EVD in pregnant women compared with non-pregnant women. p: p-value associated with Cochran's Q for heterogeneity; events: number of deaths in the group of interest; total: total number of pregnant or non-pregnant women included in the analysis.

Three studies provided sufficient information to estimate the odds of death in pregnant women compared with non-pregnant women.^34,39,44^ The pooled OR was 1.18 (95% CI 0.59 to 2.35, I^2^=31.0%, p=0.230; Figure 3).

**Figure 4.**
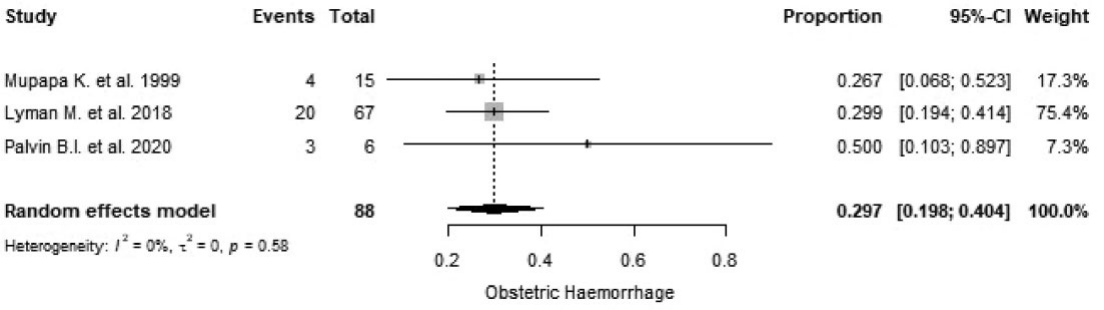
Proportional meta-analysis of studies reporting obstetric haemorrhage in Ebola-infected pregnant women. p: p-value associated with Cochran's Q for heterogeneity; events: number of women with obstetric haemorrhage; total: total number of pregnant women included in the analysis.

Obstetric haemorrhage was reported in five studies,^29,36,41,44,47^ three of which were included in a meta-analysis, resulting in an estimated absolute risk of obstetric haemorrhage in maternal EVD of 29.7% (95% CI 19.8 to 40.4, I^2^=0.0%, p=0.58; Figure 4).

### Perinatal outcomes of maternal Ebola virus infection

**Figure 5.**
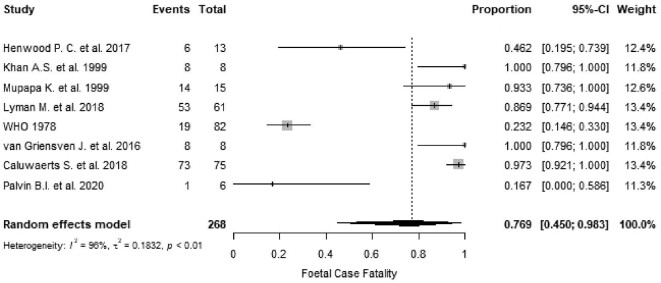
Proportional meta-analysis of studies reporting foetal loss from EVD in pregnancy. p: p-value associated with Cochran's Q for heterogeneity; events: number of foetal losses; total: total number of pregnant women included in the analysis.

Perinatal outcome measures were not defined in the included studies, and most studies did not report gestational age at which the outcomes occurred. The weighted summary estimate for foetal loss was 76.9% (95% CI 45.0 to 98.3, I^2^=96%, p<0.01; Figure 5).

**Figure 6.**
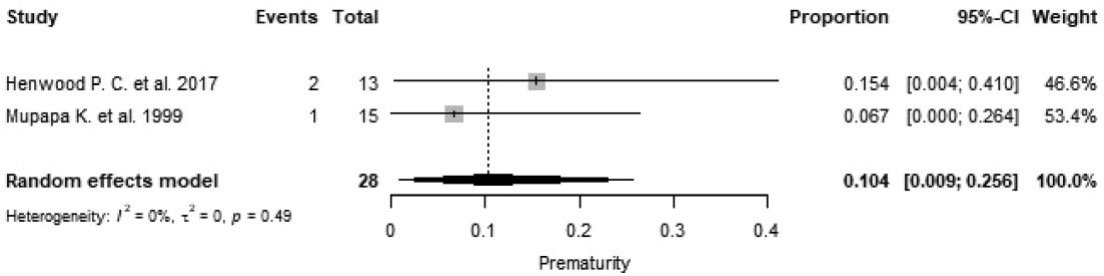
Proportional meta-analysis of studies reporting prematurity from Ebola virus disease in pregnancy. p: p-value associated with Cochran's Q for heterogeneity; events: number of preterm births; total: total number of pregnant women included in the analysis.

Prematurity was reported in six pregnant women in five studies,^29,36,39,44,50^ resulting in a pooled estimate of 10.4% (95% CI 0.9 to 25.6, I^2^=0.0%, p=0.49; Figure 6), with a mean gestational age of 31.2 weeks reported in five pregnant women.^29,36,39,44^

**Figure 7.**
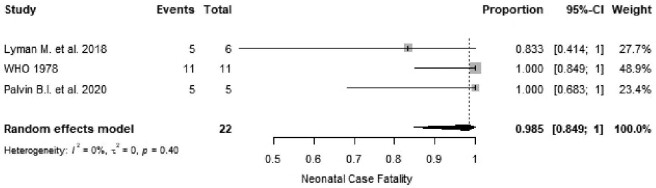
Proportional meta-analysis of studies reporting neonatal death from EVD. p: p-value associated with Cochran's Q for heterogeneity; events: number of neonatal deaths; total: total number of live births included in the analysis.

As of November 2020, only two neonates had been reported as survivors of maternal EVD.^31,36,41^ One of these neonates was diagnosed with EVD,^36^ but the other reportedly had a negative polymerase chain reaction (PCR) result.^31^ The weighted summary proportion for neonatal death was 98.5% (95% CI 84.9 to 100, I^2^=0.0%, p=0.40; Figure 7), with a median age at death of 9 d (range 0–19).^28,34,37–39,43,44,47,52^

Clinical features of Ebola-infected neonates were reported in 14 neonates in four studies^36,43,44,52^ and included fever (10/14),^36,43,44,52^ respiratory failure (1/14),^43^ coma (1/14),^43^ bleeding (1/14)^43^ and seizures (1/14).^36^ Vertical transmission of Ebola was evaluated in 24 neonates,^29,31,32,36,37,43,45–47,53,54^ with infection confirmed in 23 neonates^29,32,36,37,43,45–47,53,54^ via PCR from cord blood, foetal swabs, meconium, amniotic fluid or placental swabs. There were no reports of other complications in the reviewed literature.

### Clinical management practices for maternal Ebola virus infection

Most of the included studies did not report the use of antivirals, immunosuppressive therapy or immune modulators for the management of EVD in pregnant women. Favipiravir was used in a single case report,^36^ while convalescent plasma (CP) was used in the non-randomised trial.^49^ Of the eight pregnant patients who received CP, six of them survived. None of the studies assessed the efficacy or effectiveness of antivirals or other therapeutics in pregnant women with EVD.

Obstetric management of pregnant EVD women was not described in most studies. Termination of pregnancy was offered in two studies,^39,54^ but only one woman agreed to termination, which occurred at 7 weeks of gestation.^39^ Induced delivery, reported in six studies,^32,39,45–47,54^ generally occurred as a result of foetal demise and not necessarily due to Ebola infection. Dilatation and curettage was performed in three pregnant women^44^ due to incomplete abortion, but only one of these women survived.

Neonatal management was described in one neonate and included monoclonal antibodies (ZMapp),^36^ antivirals (GS-5734)^36^ and admission to the neonatal intensive care unit (NICU).^36^

### Sensitivity analysis and meta-regression

A post hoc sensitivity analysis dropping studies with 0% and 100% proportions and studies with <10 pregnant women did not have a substantial impact on the estimates for foetal loss and maternal death, which became 73.5% (95% CI 34.0 to 99.0, I^2^=97%, p<0.01) and 75.3% (95% CI 55.8 to 90.7, I^2^=89%, p<0.01), respectively. Sensitivity analysis was not performed for other outcomes because only one study qualified.

A meta-regression evaluating the combined effect of the sample size, the year in which the outbreak occurred, country and study design on the pooled estimates for maternal death suggests that these variables accounted for the observed heterogeneity (R^2^=100%, p<0.001), with most of the heterogeneity being as a result of the country (75.4%). Interestingly, for foetal loss these variables did not significantly account for the heterogeneity observed (p=0.629). Other potential confounders such as viral strain were included in the review, but all studies in the meta-regression model investigated viruses of the *Zaire ebolavirus* strain, and there were no data on subtypes, so further analysis could not be performed.

Given the developments in therapeutics and vaccines for EVD, we performed a subgroup analysis accounting for the year in which the outbreak occurred, considering only studies with >10 pregnant women. We found that the weighted summary of maternal death due to EVD prior to 2000 was 90.4% (95% CI 83.2 to 95.9, I^2^=0.0%, p=0.78), while after the year 2000 the estimate for maternal case fatality was 64.8% (95% CI 35.4 to 89.4, I^2^=91%, p<0.01). Foetal case fatality was 59.4% (95% CI 0.4 to 100, I^2^=97%, p<0.01) before the year 2000 and 83.5% (95% CI 57.6 to 99.0, I^2^=90.0%, p<0.01) after the year 2000.

### Publication bias for EVD

Funnel plots showed some asymmetry (Supplementary Figure 1), however, Peter's test showed no evidence of publication bias (p>0.1). The p-value for maternal case fatality proportion was 0.485, for maternal OR for pregnant women compared with non-pregnant women it was 0.727, for the foetal case fatality proportion it was 0.569 and for clinical features the p-value ranged from 0.362 to 0.941.

### Risk of bias assessment for EVD

**Figure 8.**
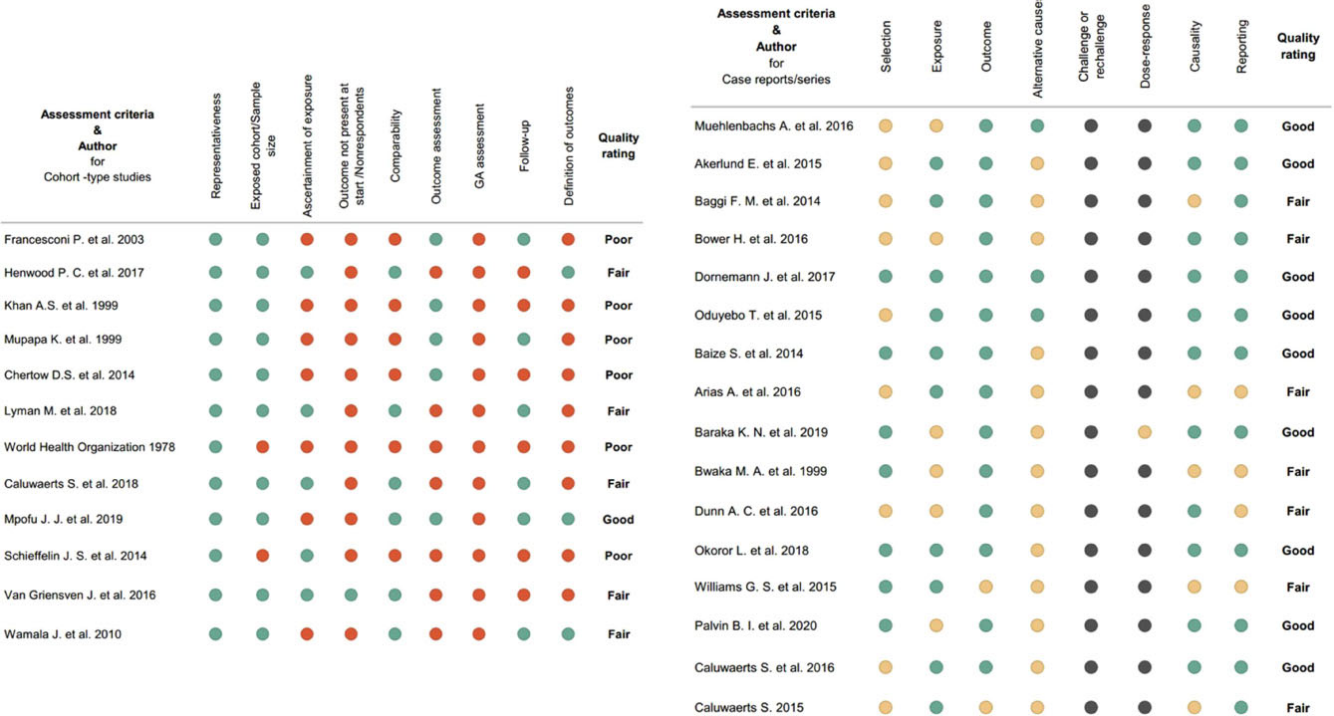
Risk of bias assessment of studies included in the systematic review and meta-analysis on EVD in pregnancy. For cohort type studies green indicates the study received a star (^∗^) for the indicated or criteria while red denotes the study didnot receive any star for that criteria on the Newcastle-Ottawa scale; for case series or case reports: green, yes; red, no; yellow, unclear/unsure; black, not applicable. GA: gestational age.

The overall risk of bias among the included case reports and case series studies was low, while the risk of bias in cohort-type studies was moderate or good in 5 of the 11 cohort-type studies. The risk of bias scores are summarised in Figure 8.

### Gap analysis for EVD

A formal analysis of potential research gaps in the grey and peer-reviewed literature suggests that, particularly for non-fatal perinatal outcomes, as well as the clinical course of EVD in pregnant women and newborns and for management of EVD in pregnancy, data are limited or non-existent (Table 3).

**Table 3.** Results of the research gap analysis for EVD in pregnancy

Gap criteria	Outcome	Justification
A	Other maternal complications of EVD	Most data on maternal complications of EVD were on mortality. There were limited or no data on other maternal complications of EVD
A	Other perinatal complications of EVD	Small sample sizes and few studies in the meta-analysis for prematurity. Most studies on vertical transmission were case reports or case series studies and other perinatal complications such as birth defects or low birthweight were almost never investigated
A	Definition of outcomes	In almost all studies outcomes were not defined
A	Trimester of pregnancy and gestational age estimation methods	Gestational ages and the methods used to estimate gestational ages were rarely reported and when recorded are subjective and often do not indicate the gestational ages at which different outcomes occurred
A	Co-infections and comorbidities	There was little or no information on comorbidities and co-infections and their effects on the clinical course of disease in pregnant women
A	Management of neonates with EVD	Management is described in one neonate with confirmed EVD
A	Management of pregnant mother with EVD	Insufficient data to evaluate the efficacy of different therapeutics for management of maternal EVD
B	Foetal losses due to EVD	Meta-analysis shows wide CIs with high heterogeneity and insufficient data to assess other potential confounders
B	Neonatal deaths due to EVD	Small sample sizes and very few studies included in meta-analysis; importantly, there is a need for evidence on mechanisms by which neonatal infection occurs
B	Clinical features of EVD in pregnancy	Meta-analysis done for most clinical features; however, the estimates have high heterogeneity with wide CIs. There were very few studies included in the meta-analysis and some studies included had a moderate to high risk of methodological bias
B	Maternal mortality—absolute risk	Meta-analysis showed wide CIs with high heterogeneity and some of the studies included had a moderate to high risk of methodological bias
C	Maternal mortality—relative risk	Estimate was precise with narrow CIs and a low heterogeneity, however, studies included had a moderate to high risk of bias
D	Clinical course of infection in pregnant women	The clinical course of EVD in pregnancy was rarely reported and most of the studies were case reports or case series studies
D	Clinical features and complications in newborns	Clinical features of neonates born to mothers with EVD were described in four studies, but most of these were case reports and case series studies

The gap analysis evaluates gaps in the literature based on expected outcomes of the review. The table summarises the gaps identified and provides a justification for each.

A: no data or insufficient information; B: meta-analysis conducted, but there were few studies in the meta-analysis, studies included have small sample sizes or the meta-analysis is associated with high heterogeneity and/or wide or extremely wide CIs. C: some of the studies included in the meta-analysis have a low-quality rating (i.e. a moderate or high risk of methodological bias); D: most of the studies discussing a specific outcome are case report or case series studies.

While meta-analyses were conducted for most of the outcomes identified in the literature, many of these estimates were imprecise, with large between-study heterogeneity, with some outcomes only discussed in case reports or case series studies. Additionally, when the estimates were precise, the sample sizes were small, and most of the cohort-type studies had a moderate to high risk of bias, which limits the strength of the available evidence.

Gestational ages were mostly self-reported and the gestational ages at which different outcomes occurred were rarely reported, as such evaluating outcomes by the trimester of pregnancy was not possible for most outcomes. Evidence synthesis was further limited by a lack of outcome definitions.

## Discussion

This review shows that while EVD is associated with a high maternal mortality rate (67.8%), the relative risk of mortality among pregnant women was not significantly different from that in non-pregnant women (OR 1.18 [95% CI 0.59 to 2.35], I^2^=31.0%, p=0.230); however, given that there are few studies in the meta-analysis and a single low-quality study^34^ accounted for the highest weighting in the meta-analysis, the evidence is inconclusive. When compared with previous studies, the estimates for maternal mortality were not significantly different. Foeller et al.,^11^ in a review published in July 2020, estimated an aggregated absolute risk of 72% and suggested that the mortality in pregnant women was not higher than that in non-pregnant women. A subgroup analysis accounting for the year in which the outbreak occurred showed that the absolute risk of maternal mortality was lower after 2000 (64.8%) compared with before 2000 (>90%) and may reflect the changes in public health preparedness. Interestingly, there was a large amount of heterogeneity in studies conducted after 2000, and this heterogeneity could perhaps be explained by differences in national responses, given that our meta-regression analysis suggests that the country in which patients were found accounted for most of the observed heterogeneity. This finding is similar to a recent review by Kawuki et al.^55^ that found the country was a possible explanatory factor for variability in EVD outcomes and underscores the need for sharing of lessons learned both within and between countries.

The absolute risk of foetal loss was high, at 76.9%, and consistent with previous reports.^9,10^ The subgroup analysis showed that unlike maternal case fatality, foetal losses after the year 2000 (83.5%) were much higher than before the year 2000 (59.4%). This may be due to improved reporting, and this finding may have been clarified by a comparison with data from the 2018–2020 DRC outbreaks, but three attempts to get unpublished data on pregnancy outcomes from that outbreak were unsuccessful. The absolute risk of neonatal deaths was high, at 98.5%, and the estimate seems precise, with low heterogeneity (95% CI 84.9 to 100, I^2^=0.0%, p=0.40), which supports the consensus that EVD almost always results in neonatal death.^9,10^ However, this meta-analysis had only three studies with an overall small size; as such, the estimate for neonatal death is not conclusive.

The high maternal and perinatal mortality seen among EVD patients supports the urgent need for preventative and therapeutic solutions, and improved screening, care and follow-up of pregnant women and newborns during outbreaks, both within and outside the treatment units, to ensure early identification and management of pregnant women and their newborns.

A lot of progress has been made in the last 6 y following the West African Ebola epidemic.^7^ Nonetheless, the review shows that there are still major gaps in the epidemiology of EVD in pregnancy, particularly with respect to non-fatal perinatal outcomes, the clinical course and the management of EVD in pregnancy. This underscores the need for well-conducted prospective cohort studies and clinical trials with pregnant women as a ‘subgroup of interest’.^14^ Given the sporadic nature of Ebola outbreaks, these gaps will be best addressed during future epidemics and would benefit from pre-epidemic preparedness for clinical research with pre-prepared protocols,^56^ improved capacity for conducting clinical research in outbreaks in at-risk countries and the inclusion of pregnant women in clinical research. Additionally, improved data quality through either harmonised databases or core datasets and outcome sets for Ebola in pregnancy will improve the evidence base for EVD.^57,58^

A common limitation across all meta-analyses performed was that they all had <10 studies, and for most outcomes the summary estimates were associated with large amounts of heterogeneity. A random effects model was used and a meta-regression and sensitivity analysis were performed to explain the heterogeneity. However, the meta-regression analyses need to be interpreted with caution, given that we have <10 studies in each meta-analysis. The reason for this is that small sample sizes (<10 studies) tend to decrease the statistical power of the meta-regression analysis. Similarly, the tests for publication bias need to be interpreted with caution given that our meta-analysis had very few studies.

## Conclusions

Maternal deaths and foetal losses in EVD are high, with almost all births to EVD pregnant women culminating in death. This evidence underscores the urgent need for preventative and therapeutic solutions, enhanced screening to identify pregnant women during outbreaks and improved care and follow-up of pregnant women and their newborns in the event of outbreaks. There is not enough evidence to conclusively rule out pregnancy as a risk factor for mortality in EVD patients, and given the paucity of evidence in pregnancy, it supports the need for robust clinical trials and prospective studies with pregnant women as a subgroup of interest.

## Supplementary Material

trab180_Supplemental_File

## Data Availability

All data generated or analysed during this study are included in this published article [and its supplementary files].
