# Supplementary material for: Ebola virus disease in pregnancy: a systematic review and meta-analysis
Source: Trans R Soc Trop Med Hyg. 2021 Dec 2;116(6):509–22. doi: 10.1093/trstmh/trab180 (PMC9157681; doi:10.1093/trstmh/trab180)
Supplement: trab180_Supplemental_File [file trab180_supplemental_file.zip › supplementary figure_publication bias.docx]

**Publication bias funnel plots for Ebola virus disease**

**Supplementary Figure:** Funnel plots for maternal case fatality and foetal loss.

A: Funnel plot of point estimates of the double-arcsine transformed maternal case fatality proportion with sample size as predictor

B: Funnel plot of point estimates of the double-arcsine transformed maternal case fatality proportion with standard error as predictor

C: Funnel plot of point estimates of the double-arcsine transformed foetal case fatality proportion with sample size as predictor

D: Funnel plot of point estimates of the double-arcsine transformed foetal case fatality proportion with standard error as predictor
