# Supplementary material for: Ebola virus disease in pregnancy: a systematic review and meta-analysis
Source: Trans R Soc Trop Med Hyg. 2021 Dec 2;116(6):509–22. doi: 10.1093/trstmh/trab180 (PMC9157681; doi:10.1093/trstmh/trab180)
Supplement: trab180_Supplemental_File [file trab180_supplemental_file.zip › supplementary table 1_search strategy_revised.docx]

| **Search** | **Search Terms for Lassa virus disease** |
| --- | --- |
| #1 | Ebola AND ("preterm birth" or "premature birth" or prematur*) |
| #2 | Ebola AND "small for gestational age" |
| #3 | Ebola AND "low birth weight" |
| #4 | Ebola AND stillbirth |
| #5 | Ebola AND "neonatal death" |
| #6 | Ebola AND ("vertical transmission" or "suspected infection in newborn" or "confirmed infection in new born" or "mother to child transmission") |
| #7 | Ebola AND (pregnanc* or pregnant) AND (antiviral OR ribavirin OR favipiravir OR "management strategies" OR therap* OR management* OR treatment*) |
| #8 | Ebola AND ("postpartum haemorrhage" or "postpartum hemorrhage") |
| #9 | Ebola AND "preterm labor" |
| #10 | Ebola AND "preterm labour" |
| #11 | Ebola and "premature rupture of membranes" |
| #12 | Ebola and "spontaneous abortion" |
| #13 | Ebola AND (maternal mortality or neonatal mortality or perinatal mortality) |
| #14 | Ebola AND (maternal outcome* or pregnancy outcome* or pregnanc* or pregnant OR birth outcome* or perinatal outcome* or obstetric outcome* or fetal outcome* or antenatal or neonat* or foetal outcome*) |
| #15 | Ebola AND (pregnanc* or pregnant) AND (" infant feeding" or "breast feeding" or "complementary feeding" or "mixed feeding" or "bottle feeding") |
| #16 | Ebola AND (pregnanc* or pregnant) AND ( "caesarean delivery" or caesarean or "instrumental vaginal delivery" or "assisted vaginal delivery" or "operative delivery" or "spontaneous vaginal delivery" or "forceps delivery" or "vacuum delivery") |
| #17 | Ebola AND (pregnanc* or pregnant) AND ("medical termination of pregnancy" or abortion) |
| #18 | Ebola AND (pregnanc* or pregnant) AND ("blood products" or platelets or "whole blood") |
| #19 | Ebola AND (pregnanc* or pregnant) AND (immunetherapy or immunotherapy or steroid* or "immunosuppressive therapy" or "convalescent plasma") |
| #20 | Ebola * AND ("preterm birth" or "premature birth" or prematur*) |
| #21 | Ebola * AND "small for gestational age" |
| #22 | Ebola * AND "low birth weight" |
| #23 | Ebola * AND stillbirth |
| #24 | Ebola * AND "neonatal death" |
| #25 | Ebola * AND ("vertical transmission" or "suspected infection in newborn" or "confirmed infection in newborn" or "mother to child transmission") |
| #26 | Ebola * AND (pregnanc* or pregnant) AND (antiviral OR ribavirin OR favipiravir OR "management strategies" OR therap* OR management* OR treatment*) |
| #27 | Ebola * AND ("postpartum haemorrhage" or "postpartum hemorrhage") |
| #28 | Ebola * AND "preterm labor" |
| #29 | Ebola * AND "preterm labour" |
| #30 | Ebola and AND (congenital malformation or congenital abnormalit* or anomal*) |
| #31 | Ebola * AND (congenital malformation or congenital abnormalit* or anomal*) AND (congenital malformation or congenital abnormalit* or anomal*) |
| #32 | Ebola and AND ("intrauterine growth restriction" or "intrauterine growth retardation") AND ("intrauterine growth restriction" or "intrauterine growth retardation") |
| #33 | Ebola * and pregnancy and "clinical features" or "clinical characteristics" or " clinical presentation" or sign* or symptom* OR Ebola and and pregnancy and "clinical features" or "clinical characteristics" or " clinical presentation" or sign* or symptom* |
| #34 | Ebola * and pregnant* OR (Ebola and and pregnant* OR Ebola * AND pregnan* |

| **Search** | **Sample of search terms for other VHFs included in the review** | |  |
| --- | --- | --- | --- |
| #1 | Lassa AND ("preterm birth" or "premature birth" or prematur*) | |  |
| #2 | Lassa AND "small for gestational age" | |  |
| #3 | Lassa AND "low birth weight" | |  |
| #4 | Lassa AND stillbirth | |  |
| #5 | Lassa AND "neonatal death" | |  |
| #6 | Lassa AND ("vertical transmission" or "suspected infection in newborn" or "confirmed infection in new born" or "mother to child transmission") | |  |
| #7 | Lassa AND (pregnanc* or pregnant) AND (antiviral OR ribavirin OR favipiravir OR "management strategies" OR therap* OR management* OR treatment*) | |  |
| #8 | Lassa AND ("postpartum haemorrhage" or "postpartum hemorrhage") | |  |
| #9 | Lassa AND "preterm labor" | |  |
| #10 | Lassa AND "preterm labour" | |  |
| #11 | Lassa and "premature rupture of membranes" | |  |
| #12 | Lassa and "spontaneous abortion" | |  |
| #13 | Lassa AND (maternal mortality or neonatal mortality or perinatal mortality) | |  |
| #14 | Lassa AND (maternal outcome* or pregnancy outcome* or pregnanc* or pregnant OR birth outcome* or perinatal outcome* or obstetric outcome* or fetal outcome* or antenatal or neonat* or foetal outcome*) | |  |
| #15 | Lassa AND (pregnanc* or pregnant) AND (" infant feeding" or "breast feeding" or "complementary feeding" or "mixed feeding" or "bottle feeding") | |  |
| #16 | Lassa AND (pregnanc* or pregnant) AND ( "caesarean delivery" or caesarean or "instrumental vaginal delivery" or "assisted vaginal delivery" or "operative delivery" or "spontaneous vaginal delivery" or "forceps delivery" or "vacuum delivery") | |  |
| #17 | Lassa AND (pregnanc* or pregnant) AND ("medical termination of pregnancy" or abortion) | |  |
| #18 | Lassa AND (pregnanc* or pregnant) AND ("blood products" or platelets or "whole blood") | |  |
| #19 | Lassa AND (pregnanc* or pregnant) AND (immunetherapy or immunotherapy or steroid* or "immunosuppressive therapy" or "convalescent plasma") | |  |
| #20 | Lassa * AND ("preterm birth" or "premature birth" or prematur*) | |  |
| #21 | Lassa * AND "small for gestational age" | |  |
| #22 | Lassa * AND "low birth weight" | |  |
| #23 | Lassa * AND stillbirth | |  |
| #24 | Lassa * AND "neonatal death" | |  |
| #25 | Lassa * AND ("vertical transmission" or "suspected infection in newborn" or "confirmed infection in newborn" or "mother to child transmission") | |  |
| #26 | Lassa * AND (pregnanc* or pregnant) AND (antiviral OR ribavirin OR favipiravir OR "management strategies" OR therap* OR management* OR treatment*) | |  |
| #27 | Lassa * AND ("postpartum haemorrhage" or "postpartum hemorrhage") | |  |
| #28 | Lassa * AND "preterm labor" | |  |
| #29 | Lassa * AND "preterm labour" | |  |
| #30 | Lassa and AND (congenital malformation or congenital abnormalit* or anomal*) | |  |
| #31 | Lassa * AND (congenital malformation or congenital abnormalit* or anomal*) AND (congenital malformation or congenital abnormalit* or anomal*) | |  |
| #32 | Lassa and AND ("intrauterine growth restriction" or "intrauterine growth retardation") AND ("intrauterine growth restriction" or "intrauterine growth retardation") | |  |
| #33 | Lassa * and pregnancy and "clinical features" or "clinical characteristics" or " clinical presentation" or sign* or symptom* OR Lassa and and pregnancy and "clinical features" or "clinical characteristics" or " clinical presentation" or sign* or symptom* | |  |
| #34 | Lassa * and pregnant* OR (Lassa and and pregnant* OR Lassa * AND pregnan* | |  |
| #1 | Crimean Congo AND ("preterm birth" or "premature birth" or prematur*) | |  |
| #2 | Crimean Congo AND "small for gestational age" | |  |
| #3 | Crimean Congo AND "low birth weight" | |  |
| #4 | Crimean Congo AND stillbirth | |  |
| #5 | Crimean Congo AND "neonatal death" | |  |
| #6 | Crimean Congo AND ("vertical transmission" or "suspected infection in newborn" or "confirmed infection in new born" or "mother to child transmission") | |  |
| #7 | | Crimean Congo AND (pregnanc* or pregnant) AND (antiviral OR ribavirin OR favipiravir OR "management strategies" OR therap* OR management* OR treatment*) | |
| #8 | | Crimean Congo AND ("postpartum haemorrhage" or "postpartum hemorrhage") | |
| #9 | | Crimean Congo AND "preterm labor" | |
| #10 | | Crimean Congo AND "preterm labour" | |
| #11 | | Crimean Congo and "premature rupture of membranes" | |
| #12 | | Crimean Congo and "spontaneous abortion" | |
| #13 | | Crimean Congo AND (maternal mortality or neonatal mortality or perinatal mortality) | |
| #14 | | Crimean Congo AND (maternal outcome* or pregnancy outcome* or pregnanc* or pregnant OR birth outcome* or perinatal outcome* or obstetric outcome* or fetal outcome* or antenatal or neonat* or foetal outcome*) | |
| #15 | | Crimean Congo AND (pregnanc* or pregnant) AND (" infant feeding" or "breast feeding" or "complementary feeding" or "mixed feeding" or "bottle feeding") | |
| #16 | | Crimean Congo AND (pregnanc* or pregnant) AND ( "caesarean delivery" or caesarean or "instrumental vaginal delivery" or "assisted vaginal delivery" or "operative delivery" or "spontaneous vaginal delivery" or "forceps delivery" or "vacuum delivery") | |
| #17 | | Crimean Congo AND (pregnanc* or pregnant) AND ("medical termination of pregnancy" or abortion) | |
| #18 | | Crimean Congo AND (pregnanc* or pregnant) AND ("blood products" or platelets or "whole blood") | |
| #19 | | Crimean Congo AND (pregnanc* or pregnant) AND (immunetherapy or immunotherapy or steroid* or "immunosuppressive therapy" or "convalescent plasma") | |
| #20 | | Crimean Congo * AND ("preterm birth" or "premature birth" or prematur*) | |
| #21 | | Crimean Congo * AND "small for gestational age" | |
| #22 | | Crimean Congo * AND "low birth weight" | |
| #23 | | Crimean Congo * AND stillbirth | |
| #24 | | Crimean Congo * AND "neonatal death" | |
| #25 | | Crimean Congo * AND ("vertical transmission" or "suspected infection in newborn" or "confirmed infection in newborn" or "mother to child transmission") | |
| #26 | | Crimean Congo * AND (pregnanc* or pregnant) AND (antiviral OR ribavirin OR favipiravir OR "management strategies" OR therap* OR management* OR treatment*) | |
| #27 | | Crimean Congo * AND ("postpartum haemorrhage" or "postpartum hemorrhage") | |
| #28 | | Crimean Congo * AND "preterm labor" | |
| #29 | | Crimean Congo * AND "preterm labour" | |
| #30 | | Crimean Congo and AND (congenital malformation or congenital abnormalit* or anomal*) | |
| #31 | | Crimean Congo * AND (congenital malformation or congenital abnormalit* or anomal*) AND (congenital malformation or congenital abnormalit* or anomal*) | |
| #32 | | Crimean Congo and AND ("intrauterine growth restriction" or "intrauterine growth retardation") AND ("intrauterine growth restriction" or "intrauterine growth retardation") | |
| #33 | | Crimean Congo * and pregnancy and "clinical features" or "clinical characteristics" or " clinical presentation" or sign* or symptom* OR Crimean Congo and and pregnancy and "clinical features" or "clinical characteristics" or " clinical presentation" or sign* or symptom* | |
| #34 | | Crimean Congo * and pregnant* OR (Crimean Congo and and pregnant* OR Crimean Congo * AND pregnan* | |
| #1 | | Rift Valley AND ("preterm birth" or "premature birth" or prematur*) | |
| #2 | | Rift Valley AND "small for gestational age" | |
| #3 | | Rift Valley AND "low birth weight" | |
| #4 | | Rift Valley AND stillbirth | |
| #5 | | Rift Valley AND "neonatal death" | |
| #6 | | Rift Valley AND ("vertical transmission" or "suspected infection in newborn" or "confirmed infection in new born" or "mother to child transmission") | |
| #7 | | Rift Valley AND (pregnanc* or pregnant) AND (antiviral OR ribavirin OR favipiravir OR "management strategies" OR therap* OR management* OR treatment*) | |
| #8 | | Rift Valley AND ("postpartum haemorrhage" or "postpartum hemorrhage") | |
| #9 | | Rift Valley AND "preterm labor" | |
| #10 | | Rift Valley AND "preterm labour" | |
| #11 | | Rift Valley and "premature rupture of membranes" | |
| #12 | | Rift Valley and "spontaneous abortion" | |
| #13 | | Rift Valley AND (maternal mortality or neonatal mortality or perinatal mortality) | |
| #14 | | Rift Valley AND (maternal outcome* or pregnancy outcome* or pregnanc* or pregnant OR birth outcome* or perinatal outcome* or obstetric outcome* or fetal outcome* or antenatal or neonat* or foetal outcome*) | |
| #15 | | Rift Valley AND (pregnanc* or pregnant) AND (" infant feeding" or "breast feeding" or "complementary feeding" or "mixed feeding" or "bottle feeding") | |
| #16 | | Rift Valley AND (pregnanc* or pregnant) AND ( "caesarean delivery" or caesarean or "instrumental vaginal delivery" or "assisted vaginal delivery" or "operative delivery" or "spontaneous vaginal delivery" or "forceps delivery" or "vacuum delivery") | |
| #17 | | Rift Valley AND (pregnanc* or pregnant) AND ("medical termination of pregnancy" or abortion) | |
| #18 | | Rift Valley AND (pregnanc* or pregnant) AND ("blood products" or platelets or "whole blood") | |
| #19 | | Rift Valley AND (pregnanc* or pregnant) AND (immunetherapy or immunotherapy or steroid* or "immunosuppressive therapy" or "convalescent plasma") | |
| #20 | | Rift Valley * AND ("preterm birth" or "premature birth" or prematur*) | |
| #21 | | Rift Valley * AND "small for gestational age" | |
| #22 | | Rift Valley * AND "low birth weight" | |
| #23 | | Rift Valley * AND stillbirth | |
| #24 | | Rift Valley * AND "neonatal death" | |
| #25 | | Rift Valley * AND ("vertical transmission" or "suspected infection in newborn" or "confirmed infection in newborn" or "mother to child transmission") | |
| #26 | | Rift Valley * AND (pregnanc* or pregnant) AND (antiviral OR ribavirin OR favipiravir OR "management strategies" OR therap* OR management* OR treatment*) | |
| #27 | | Rift Valley * AND ("postpartum haemorrhage" or "postpartum hemorrhage") | |
| #28 | | Rift Valley * AND "preterm labor" | |
| #29 | | Rift Valley * AND "preterm labour" | |
| #30 | | Rift Valley and AND (congenital malformation or congenital abnormalit* or anomal*) | |
| #31 | | Rift Valley * AND (congenital malformation or congenital abnormalit* or anomal*) AND (congenital malformation or congenital abnormalit* or anomal*) | |
| #32 | | Rift Valley and AND ("intrauterine growth restriction" or "intrauterine growth retardation") AND ("intrauterine growth restriction" or "intrauterine growth retardation") | |
| #33 | | Rift Valley * and pregnancy and "clinical features" or "clinical characteristics" or " clinical presentation" or sign* or symptom* OR Rift Valley and and pregnancy and "clinical features" or "clinical characteristics" or " clinical presentation" or sign* or symptom* | |
| #34 | | Rift Valley * and pregnant* OR (Rift Valley and and pregnant* OR Rift Valley * AND pregnan* | |
| #1 | | Marburg AND ("preterm birth" or "premature birth" or prematur*) | |
| #2 | | Marburg AND "small for gestational age" | |
| #3 | | Marburg AND "low birth weight" | |
| #4 | | Marburg AND stillbirth | |
| #5 | | Marburg AND "neonatal death" | |
| #6 | | Marburg AND ("vertical transmission" or "suspected infection in newborn" or "confirmed infection in new born" or "mother to child transmission") | |
| #7 | | Marburg AND (pregnanc* or pregnant) AND (antiviral OR ribavirin OR favipiravir OR "management strategies" OR therap* OR management* OR treatment*) | |
| #8 | | Marburg AND ("postpartum haemorrhage" or "postpartum hemorrhage") | |
| #9 | | Marburg AND "preterm labor" | |
| #10 | | Marburg AND "preterm labour" | |
| #11 | | Marburg and "premature rupture of membranes" | |
| #12 | | Marburg and "spontaneous abortion" | |
| #13 | | Marburg AND (maternal mortality or neonatal mortality or perinatal mortality) | |
| #14 | | Marburg AND (maternal outcome* or pregnancy outcome* or pregnanc* or pregnant OR birth outcome* or perinatal outcome* or obstetric outcome* or fetal outcome* or antenatal or neonat* or foetal outcome*) | |
| #15 | | Marburg AND (pregnanc* or pregnant) AND (" infant feeding" or "breast feeding" or "complementary feeding" or "mixed feeding" or "bottle feeding") | |
| #16 | | Marburg AND (pregnanc* or pregnant) AND ( "caesarean delivery" or caesarean or "instrumental vaginal delivery" or "assisted vaginal delivery" or "operative delivery" or "spontaneous vaginal delivery" or "forceps delivery" or "vacuum delivery") | |
| #17 | | Marburg AND (pregnanc* or pregnant) AND ("medical termination of pregnancy" or abortion) | |
| #18 | | Marburg AND (pregnanc* or pregnant) AND ("blood products" or platelets or "whole blood") | |
| #19 | | Marburg AND (pregnanc* or pregnant) AND (immunetherapy or immunotherapy or steroid* or "immunosuppressive therapy" or "convalescent plasma") | |
| #20 | | Marburg * AND ("preterm birth" or "premature birth" or prematur*) | |
| #21 | | Marburg * AND "small for gestational age" | |
| #22 | | Marburg * AND "low birth weight" | |
| #23 | | Marburg * AND stillbirth | |
| #24 | | Marburg * AND "neonatal death" | |
| #25 | | Marburg * AND ("vertical transmission" or "suspected infection in newborn" or "confirmed infection in newborn" or "mother to child transmission") | |
| #26 | | Marburg * AND (pregnanc* or pregnant) AND (antiviral OR ribavirin OR favipiravir OR "management strategies" OR therap* OR management* OR treatment*) | |
| #27 | | Marburg * AND ("postpartum haemorrhage" or "postpartum hemorrhage") | |
| #28 | | Marburg * AND "preterm labor" | |
| #29 | | Marburg * AND "preterm labour" | |
| #30 | | Marburg and AND (congenital malformation or congenital abnormalit* or anomal*) | |
| #31 | | Marburg * AND (congenital malformation or congenital abnormalit* or anomal*) AND (congenital malformation or congenital abnormalit* or anomal*) | |
| #32 | | Marburg and AND ("intrauterine growth restriction" or "intrauterine growth retardation") AND ("intrauterine growth restriction" or "intrauterine growth retardation") | |
| #33 | | Marburg * and pregnancy and "clinical features" or "clinical characteristics" or " clinical presentation" or sign* or symptom* OR Marburg and and pregnancy and "clinical features" or "clinical characteristics" or " clinical presentation" or sign* or symptom* | |
| #34 | | Marburg * and pregnant* OR (Marburg and and pregnant* OR Marburg * AND pregnan* | |
